# Supplementary material for: Elevated Liver Fibrosis Progression in Isolated PSC Patients and Increased Malignancy Risk in a PSC-IBD Cohort: A Retrospective Study
Source: Int J Mol Sci. 2023 Oct 21;24(20):15431. doi: 10.3390/ijms242015431 (PMC10607359; doi:10.3390/ijms242015431)
Supplement: Supplementary file 1 [file ijms-24-15431-s001.zip › ijms-2654592-supplementary.pdf]

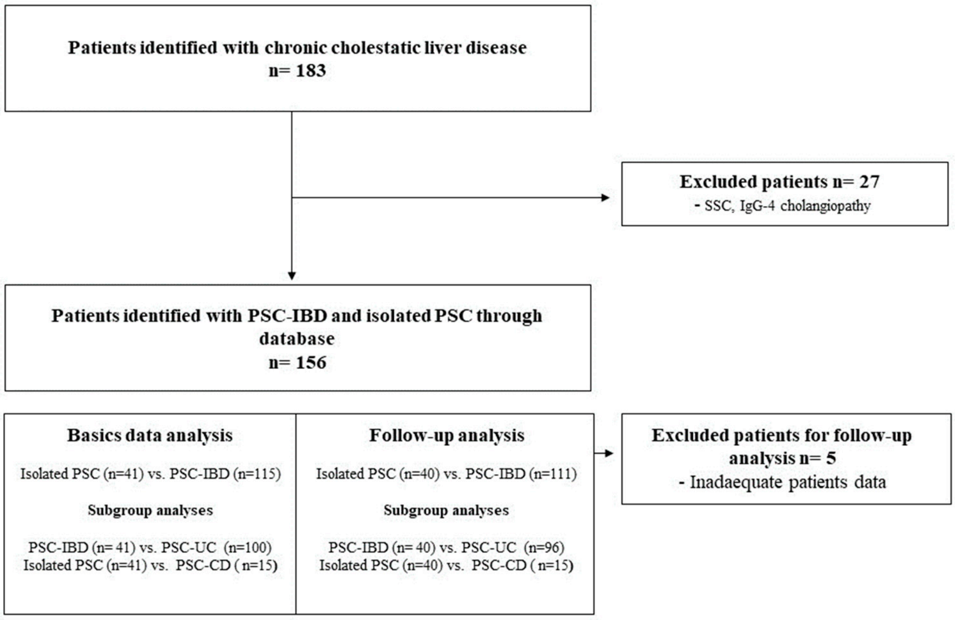

Supplemental Figure S1: Prisma diagram of the study cohort: Basic data analysis was performed on 156 patients. Follow-up analysis was performed on 151 patients. PSC, primary sclerosing cholangitis; IBD, inflammatory bowel disease; UC, ulcerative cholangitis; CD, Crohn's disease.
